# The role of invertases in plant compensatory responses to simulated herbivory

**DOI:** 10.1186/s12870-015-0655-6

**Published:** 2015-11-16

**Authors:** Madhura H. Siddappaji, Daniel R. Scholes, Sindhu M. Krishnankutty, Bernarda Calla, Steven J. Clough, Raymond E. Zielinski, Ken N. Paige

**Affiliations:** Department of Animal Biology, University of Illinois, Urbana, IL 61801 USA; Department of Biology, University of Indianapolis, Indianapolis, IN 46227 USA; Department of Entomology, University of Illinois, Urbana, IL 61801 USA; USDA-Agricultural Research Service Pacific Basin, Agricultural Research Center, Hilo, HI 96720 USA; USDA-Agricultural Research Service and the Department of Crop Sciences, University of Illinois, Urbana, IL 61801 USA; Department of Plant Biology, University of Illinois, Urbana, IL 61801 USA

**Keywords:** *Arabidopsis*, Invertase, Overcompensation, Herbivory, *G6PD1*, Sucrose, Oxidative pentose phosphate pathway

## Abstract

**Background:**

The ability of a plant to overcome animal-induced damage is referred to as compensation or tolerance and ranges from undercompensation (decreased fitness when damaged) to overcompensation (increased fitness when damaged). Although it is clear that genetic variation for compensation exists among plants, little is known about the specific genetic underpinnings leading to enhanced fitness. Our previous study identified the enzyme *GLUCOSE-6-PHOSPHATE DEHYDROGENASE 1* (*G6PD1)* as a key regulator contributing to the phenomenon of overcompensation via its role in the oxidative pentose phosphate pathway (OPPP). Apart from *G6PD1* we also identified an invertase gene which was up-regulated following damage and that potentially integrates with the OPPP. The invertase family of enzymes hydrolyze sucrose to glucose and fructose, whereby the glucose produced is shunted into the OPPP and presumably supports plant regrowth, development, and ultimately compensation. In the current study, we measured the relative expression of 12 invertase genes over the course of plant development in the *Arabidopsis thaliana* genotypes Columbia-4 and Landsberg *erecta*, which typically overcompensate and undercompensate, respectively, when damaged. We also compared the compensatory performances of a set of invertase knockout mutants to the Columbia-4 wild type.

**Results:**

We report that Columbia-4 significantly up-regulated 9 of 12 invertase genes when damaged relative to when undamaged, and ultimately overcompensated for fruit production. Landsberg *erecta*, in contrast, down-regulated two invertase genes following damage and suffered reduced fitness. Knockout mutants of two invertase genes both exhibited significant undercompensation for fruit production, exhibiting a complete reversal of the wild type Col-4’s overcompensation.

**Conclusion:**

Collectively, these results confirm that invertases are essential for not only normal plant growth and development, but also plants’ abilities to regrow and ultimately compensate for fitness following apical damage.

**Electronic supplementary material:**

The online version of this article (doi:10.1186/s12870-015-0655-6) contains supplementary material, which is available to authorized users.

## Background

While plants are generally presumed to be impacted negatively by incurring damage, considerable evidence has accumulated that demonstrates that some plants exhibit enhanced fitness when damaged (i.e. plants can overcompensate). Ecologists and evolutionary biologists first became interested in the phenomenon of overcompensation during the mid-1970s when several researchers [1–3] reported that herbivory may result in an increase, rather than a decrease, in the growth and reproductive success of some plant species [4]. These observations were initially dismissed as the result of reallocation of below-ground resources to promote the regrowth of above-ground structures, eventually resulting in a net fitness decrement over the lifetime of perennial plants [5, 6]. Paige & Whitham [7], however, provided evidence that herbivory can, under some circumstances, lead to enhanced plant fitness. Their choice of a monocarpic plant (i.e., one that reproduces only once and then dies) simplified the estimation of lifetime fitness and eliminated the possibility that apparent overcompensation came at the expense of future reproduction [7, 8]. Specifically, Paige & Whitham [7] reported that when ungulate herbivores removed 95 % or more of the above-ground biomass of the monocarpic biennial scarlet gilia, *Ipomopsis aggregata*, the product of lifetime seed production, seed germination, and seedling survival averaged 3.0 times that of the uneaten controls. This increase in relative fitness correlated strongly with changes in plant architecture—ungulate removal of scarlet gilia’s single inflorescence resulted in the production of multiple flowering stalks due to the release of apical dominance, leading to an overall increase in both above- and below-ground biomass [7, 9–12]. Many researchers have since uncovered additional examples of overcompensation in a wide variety of species and environments [13–18]. Thus, the apparently paradoxical phenomenon of overcompensation can no longer be summarily dismissed [19].

There is also evidence that compensatory performance is genetically influenced. For example, within-species variation in compensation has been reported where certain genotypes of a species overcompensate, whereas others consistently exhibit equal- or undercompensation [20–22]. The heritability of traits associated with tolerance, such as reduced phenological delay and increased branching, has even been quantified in one population of scarlet gilia [22]. In addition, studies comparing historically grazed and ungrazed populations of the plant *Gentianella campestris* indicate that repeatedly grazed populations can evolve overcompensation while ungrazed populations remain completely intolerant [16].

Although there is evidence that genetic variation for compensation exists, little is known about the genetic mechanisms leading to enhanced growth and reproduction following herbivory. In a recent study [23], we used a suite of molecular and quantitative genetic techniques for candidate gene discovery in a family of recombinant inbred lines (RILs) of *Arabidopsis thaliana* that vary in their abilities to compensate for apical damage (see also [24, 25]). Specifically, quantitative trait loci (QTL) mapping revealed three genome regions that collectively explain 48.2 % of the variation in fitness compensation within the family of RILs. Further, microarray analyses of the overcompensating genotype Columbia-4 identified a total of 109 differentially expressed genes between damaged and undamaged plants [23], one of which resides within a significant QTL region. Subsequent gene knockout and complementation methodologies collectively point to one gene with large effect on compensatory performance: *GLUCOSE-6-PHOSPHATE DEHYDROGENASE 1* (*G6PD1*, At5g35790). *G6PD1* encodes an enzyme that catalyzes the rate-limiting first step of the oxidative pentose-phosphate pathway (OPPP), which plays a central role in generalized metabolism and biosynthesis. In addition to metabolic intermediates and the reductant NADPH, the OPPP also supports the production of nucleotides and presumably endoreduplication (i.e. genome re-replication leading to increased cellular ploidy), which is known to contribute to compensatory performance through its likely effects on cell growth, gene expression, and metabolism [24–27].

Apart from *G6PD1*, our QTL and microarray analyses pointed to another gene of likely effect: *CYTOSOLIC INVERTASE 1* (At1g35580) [23]. Invertases comprise a family of metabolic isoenzymes that catalyze the hydrolysis of sucrose into glucose and fructose [28]. There are three forms of invertase *viz*., neutral/cytosolic, cell wall and vacuolar, that share similar catalytic function. The glucose produced through sucrose hydrolysis is in part shunted to *G6PD1* in the OPPP to produce ribulose-5-phosphate and erythrose-4-phosphate [29]. These compounds in turn serve as intermediates for nucleotide synthesis and plant defensive chemistry through the shikimate pathway [29–31]. Due to their catalysis of a reaction that directly feeds the rate-limiting step of the OPPP, invertases may therefore play an important role in plant compensation to damage through their integration with the OPPP and subsequent downstream metabolic processes.

In this study, we sought to determine the importance of the invertase isoenzyme family in plant compensation to apical damage using the *A. thaliana* genotypes Columbia-4 (Col-4) and Landsberg *erecta* (L*er*). Specifically, we compared a) the expression of the invertase isoenzyme family between damaged and undamaged plants of Col-4 and L*er* through development, and b) the compensatory performance of loss-of-function mutants for individual invertase genes relative to the wild type Col-4. Our results collectively suggest that invertases play an important role in determining a plant’s ability to respond to, and to even overcompensate for, apical damage, improving our understanding of the molecular basis for this long-debated phenomenon in plant-animal interactions.

## Methods

### Plant genotypes, growth conditions, and fitness measures

Seeds of the *A. thaliana* accessions Col-4, an overcompensating genotype, and Landsberg *erecta*, an undercompensating genotype, were stratified at 4 °C for 3 days to obtain uniform germination. One hundred and twenty seeds of each accession were then planted in 3 in. diameter circular pots containing Sunshine LC1 professional growing mix (Sun Gro Horticulture, Canada Ltd, Seba Beach, AB, Canada). Plants were grown in a growth chamber maintained at 24–26 °C, ~40 % relative humidity, and on a 16:8 h light:dark cycle. When elongating inflorescences (i.e. flowering stems) of half (60) of the plants of each accession reached a height of 6 cm, the inflorescences were clipped with scissors, leaving only 1 cm of remaining stem tissue. The damage imposed by this clipping regimen is comparable in severity and elicits the release of apical dominance similarly to the natural mammalian herbivory experienced by *A. thaliana* throughout its native range (Scholes et al. *personal observation*). The remaining half of the plants of each accession remained unclipped for comparison. Plants were randomly assigned to the clipping treatment (clipped or unclipped).

At the completion of senescence, total silique yield was measured for 30 plants/ accession (15 clipped and 15 unclipped). Because silique yield is highly correlated with seed yield in *A. thaliana* (see [25, 26]), we consider silique yield as our ultimate measure of plant fitness here.

### Tissue collection, RNA extraction, cDNA synthesis, and qPCR for gene expression

Plant samples for invertase gene expression analysis were collected throughout the growth and development of the plants. Tissue samples were specifically collected at 1, 5, and 15 days after clipping (DAC), and at 50 % flowering (when 50 % of the flowers are open on the plant). Rosette leaves were collected at the 1 day after clipping time point and secondary meristems were collected at the remaining time points. The rationale for collecting secondary meristems at later points in time is due to the translocation of nutrients to developing tissues (e.g. secondary meristems, cauline leaves, siliques) during later development, where invertases aid in the transport of sugars into sink cells from the phloem [28]. The 50 % flowering time point was designated as the time at which the number of flowers and the number of flower buds were approximately equivalent. Plant tissues for gene expression were immediately flash-frozen in liquid nitrogen upon collection and stored at −70 °C until processing. Tissue samples were then pooled from three plants to constitute each biological replicate for each genotype × treatment group.

Total RNA was extracted from each biological replicate using TRIzol (Life technologies, Carlsbad, CA, USA). In brief, the tissues were lysed in TRIzol, and nucleic acids were precipitated using chloroform and isopropanol, washed using 70 and 90 % ethanol, and RNA was suspended in 30 μl water. RNA samples were then treated with DNase (NEB, Cambridge, MA) to remove DNA contamination. Approximately 2 μg of RNA was reverse transcribed for cDNA library construction using the Advantage RT-for-PCR Kit (Clontech, Mountain View, CA, USA). The cDNA libraries were diluted to a concentration of 30 ng/μl, and relative quantification of invertase transcript abundance was performed using invertase gene specific primers [32] (Additional file 1: Table S1). Comparisons of primers for Columbia and Landsberg *erecta *showed no differences in sequence in nine of the twelve invertase genes. For three neutral invertase genes, Landsberg showed one SNP difference in the forward primer of At1g56560, one SNP difference in the reverse primer of At5g22510 and one SNP difference in both the forward and reverse primers of At4g09510. Amplification for all three of these invertases were of similar magnitude when comparing Columbia to Landsberg (see Figure 4), thus, the efficiency of expression was unlikely altered by the SNP. A housekeeping gene *UBIQUITIN CONJUGATING ENZYME 9* (*UBC9;* At4g27960) was used to normalize gene expression. We also used EF1-α, however, gene expression varied across tissues for this housekeeping gene, thus, only Ubiquitin was used to normalize gene expression. Invertase isoenzymes are encoded by a diverse family of genes. Random, pair-wise comparison of the coding sequences of full length invertase cDNAs (obtained for The Arabidopsis Information Resource) revealed an average number of nucleotide differences per site of 0.5533 (Clustal *X*2, University College Dublin, Dublin Ireland). This difference made possible quantitative PCR (qPCR) comparisons of the levels of expression of different invertase gene family members. Quantitative PCR (qPCR) was performed in 10 μL reactions in a 7300 Applied Biosystems thermocycler (Applied Biosystems, Foster City, CA, USA) with a final cDNA concentration of 6 ng/μL. A blank control without cDNA was included to make sure that the reagents used were not contaminated. Three biological replicates and three technical replicates were assessed for gene expression for each of the treatment × genotype × time point × gene groups. Thus, a total of 90 plants/accession were used in measuring gene expression (2 treatments X 5 time points X 9 plants/time period [3 pooled for each of the 3 biological replicates] X 2 genotypes = 180 plants total used in measuring gene expression). All plants were randomly assigned to treatments/time-points for tissue collection. The cycle threshold (Ct) values of invertase and *UBC9* were averaged across technical replicates, and then the Pfaffl method was used to calculate the relative gene expression for each biological replicate [33]. Briefly, the average Ct value of the gene of interest was subtracted from the Ct value of the housekeeping gene *UBC9* to obtain the ∆Ct for both control (unclipped) and treatment (clipped) plants. The ∆∆Ct was calculated by subtracting the treatment ∆Ct from the control ∆Ct, with the relative gene expression value defined as 2^-∆∆Ct^ [33]. It is important to point out that our clipping treatment may overly simplify effects following the removal of apical dominance. For example, proteins, hormones and microbes in the saliva of vertebrate herbivores could have large effects on gene expression in ways that are not seen in a plant’s response to manual wounding/clipping [34, 35. Our studies on scarlet gilia however, failed to show any differential phenotypic or fitness effects on plants that were naturally browsed by ungulate herbivores or plants that were experimentally clipped [7, 9]. Whether gene expression results for *Arabidopsis *would be differentially altered by natural herbivory from those of our clipping treatment will need to be addressed in future studies. 

### Invertase knockout mutants

**Fig. 1:**
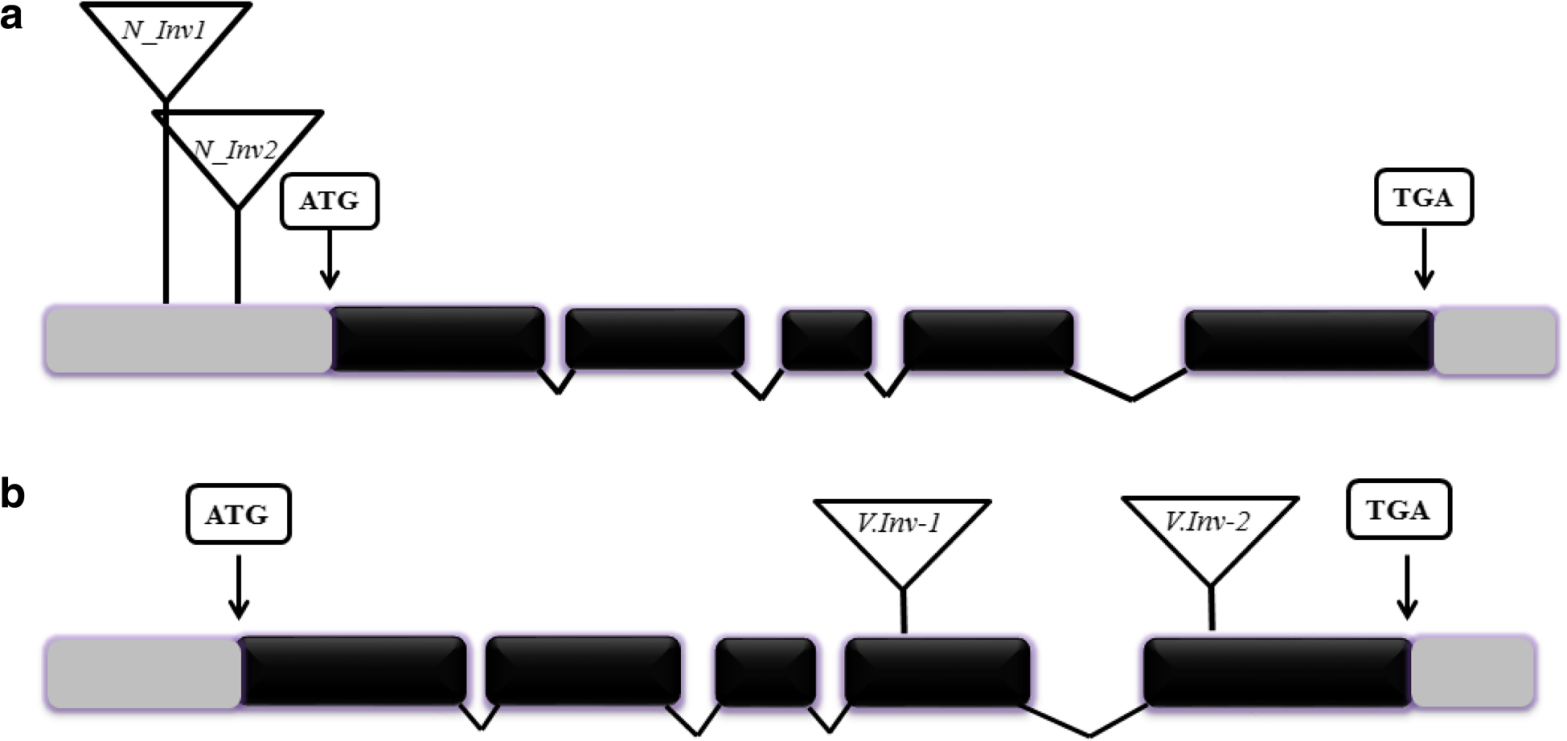
Schematic representation of **a**) neutral invertase (At4g09510) and **b**) vacuolar invertase (At1g35580) genes with the position of T-DNA insertions. Exons (dark shading), introns (lines), 5′ UTR and 3′ UTR (light shading) along with start and stop codons are shown. The T-DNA inserts are represented as inverted triangles. See Additional file 1: Table S2 for the primers used for confirming homozygosity of T-DNA insertion in knockout lines

To experimentally assess the importance of specific invertase genes on compensation, we selected a set of T-DNA invertase gene knockout mutants for assessment of compensatory performance. All knockout mutants shared the same genetic background of the overcompensating Col-4 genotype; mutants with the L*er* genetic background were not available. As we were unable to verify homozygosity of T-DNA knockout mutants for cell wall invertase isoforms, we conducted the study with mutants of one of the two vacuolar and one of the neutral invertases, each with two individual T-DNA knockout lines that differ in T-DNA insertion position (vacuolar invertase: *CINV1*, At1g35580, mutants V_Inv1 – SAIL_637_C02 and V_Inv2 – WiscDsLox450D11; neutral invertase: *CINV2*, At4G09510, NInv_1 – SAIL_441_G04 and NInv_1 – SAIL_518_D02; Fig. 1a and b). Homozygosity of T-DNA insertions was confirmed by designing primers specific to each T-DNA insertion position using the Salk priming protocol (http://signal.salk.edu/tdnaprimers.2.html; see Additional file 1: Table S2 for primers used). In addition to the invertase knockout mutants, we also selected the overcompensating Col-4 genetic background and the undercompensating genotype L*er* as wild type controls for compensation analysis. A total of 40 plants per genotype (6 genotypes including the 4 knockout mutants, L*er* and Col-4, for a total of 240 plants) were grown, half were randomly chosen and experimentally clipped and siliques were counted to assess compensation according to the procedures described above.

### Statistical analyses

Statistical analyses were conducted using Origin (v.9.0, Origin Lab Corporation, Northampton, MA, USA) and Systat (v.13.1, SYSTAT, inc. Evanston, Illinois). Two sample t-tests were used to assess clipping effects on gene expression for each given day after clipping for each of the twelve invertases and each of the two genotypes. To test the overall effect of clipping on invertase gene expression, average gene expression (of clipped and unclipped plants) through developmental time, and the interactive effect of clipping and developmental time, Ct values were compared between treatments for each genotype by two-way ANOVA (treatment as one factor and days after clipping as the second factor). The knockout data were analyzed using an analysis of variance followed by linear contrasts comparing clipped to unclipped plants within each treatment group so that we could assess whether knockout treatments altered the compensatory outcome from that of overcompensation observed in the Col-4 wild type. The compensatory performance of each genotype was determined by comparing total silique yield between unclipped and clipped plants by ANOVA.

## Results

### Apical damage induces invertase gene expression in the overcompensating genotype Columbia-4, but not in the undercompensator Landsberg *erecta*

**Fig. 2:**
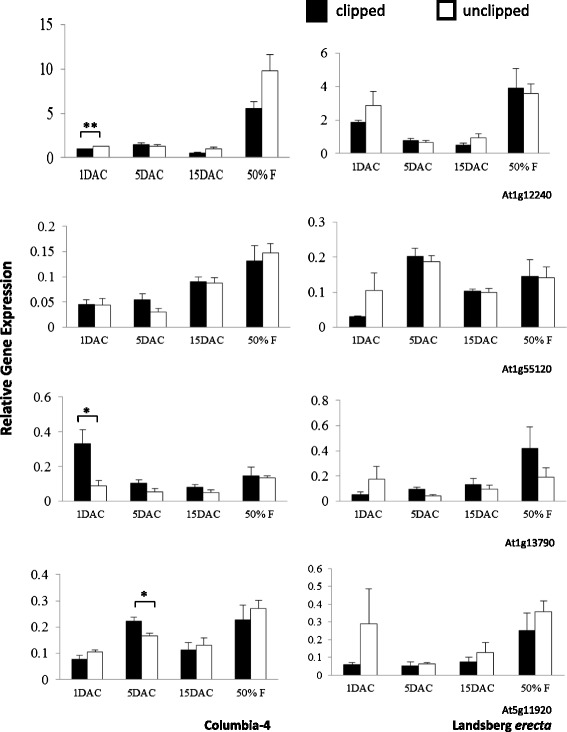
Relative expression of cell wall invertase genes through developmental time (one day before clipping not shown). Shown are relative gene expression (Ct) values with respect to the house keeping gene *UBC9*. Asterisks indicate that invertase expression differed significantly between unclipped and clipped plants at * *p* < 0.05 and ** *p* < 0.01

**Fig. 3:**
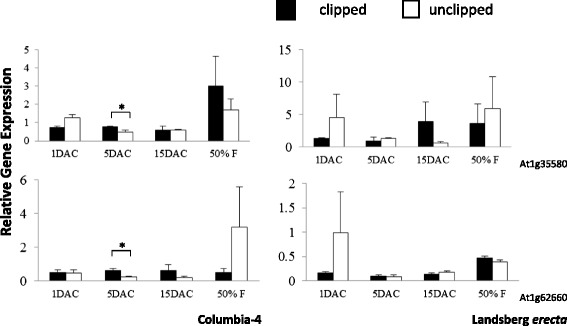
Relative expression of vacuolar invertase genes through developmental time (one day before clipping not shown). Shown are relative gene expression values with respect to the house keeping gene *UBC9*. Asterisks indicate that invertase expression differed significantly between unclipped and clipped plants at * *p* < 0.05 and ** *p* < 0.01

**Fig. 4:**
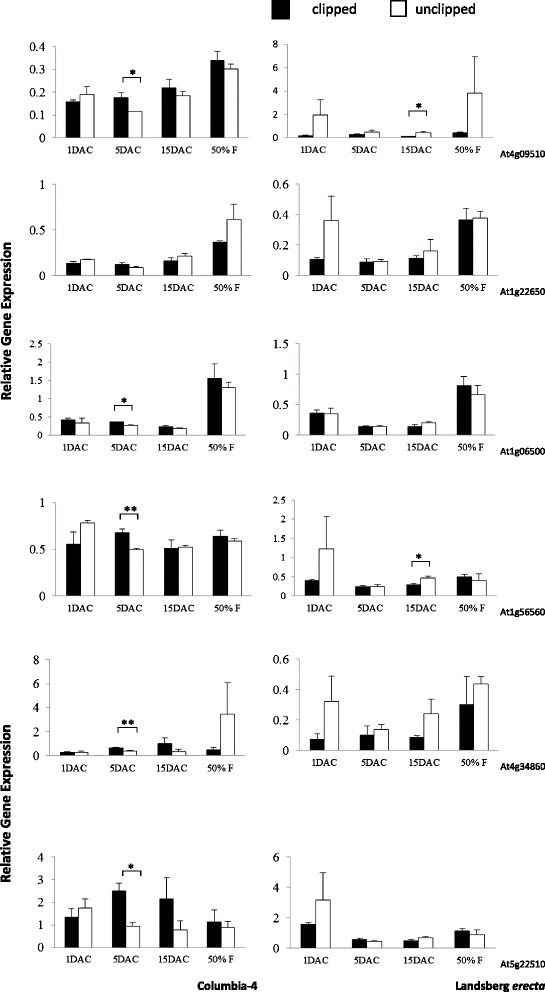
Relative expression of neutral invertase genes through developmental time (one day before clipping not shown). Shown are relative gene expression (Ct) values with respect to the house keeping gene *UBC9*. Asterisks indicate that invertase expression differed significantly between unclipped and clipped plants at * *p* < 0.05 and ** *p* < 0.01

Because previous QTL analyses revealed a potential contribution to overcompensation by at least one *Arabidopsis* invertase gene [23], we examined the expression patterns of all the members of this gene family in response to removal of the floral apex. In the overcompensating accession, Col-4, we found significant changes in invertase gene expression that were distinct from those observed in the undercompensating accession, L*er*. These changes were most prominent 1 to 5 days after clipping among certain members of the cell wall (Fig. 2), vacuolar (Fig. 3) and neutral (Fig. 4) invertase gene family. In contrast, when differences in invertase mRNA expression between clipped and unclipped plants were observed in L*er*, they occurred longer after clipping and the changes were opposite those observed in the overcompensating line.

Ten invertase genes in total displayed significant changes in expression upon clipping (clipped plants relative to unclipped plants) in Col-4 and/or L*er* for at least one time point (Figs. 2, 3 and 4, Additional file 1: Tables S3 and S4). One cell wall invertase (At3g13790) was significantly up-regulated at 1 day after clipping (DAC) in Col-4, while another (At1g12240) was down-regulated at 1 DAC. At 5 DAC, Col-4 exhibited increased expression of both vacuolar invertases (At1g35580 and At1g62660), one cell wall invertase (At5g11920), and five neutral invertases (At1g56560, At1g06500, At4g09510, At4g34860, and At5g22510; Figs. 2, 3 and 4, Additional file 1: Tables S3 and S4). Col-4 did not exhibit any significant changes in invertase gene expression at 15 DAC, however, L*er* experienced significant down-regulation of two neutral invertases (At1g56560 and At4g09510) following clipping at this time point (Fig. 4, Additional file 1: Table S4). L*er* did not experience any significant changes in invertase gene expression upon clipping at any preceding time point (1 and 5 DAC), and neither genotype experienced significant changes at the 50 % flowering time point.

In addition, one invertase (a neutral invertase, At4g34860) in L*er* and two invertases in Col-4 (a cell wall invertase, Atg12240 and a neutral invertase, Atg22650) showed significant overall cumulative differences (average values of expression across all developmental time points; i.e., Treatment effects) in gene expression between clipped and unclipped plants, with unclipped plants in both L*er* and Col-4 showing greater overall expression (Figs. 2 and 4, Additional file 1: Tables S3 and S4).

Furthermore, seven of twelve invertases in Col-4 and L*er* showed significant differences in gene expression based on developmental timing (average value of expression for clipped and unclipped plants at a given point in time)*.* Five of seven invertases in Col-4 showed higher expression at 50 % flowering relative to all preceding time-points (At1g12240, At1g55120, At1g22650, At4g09510, and At1g06500). Three of seven invertases in L*er* showed higher expression at 50 % flowering over all preceding time-points (At1g22650, At4g34860, and At1g06500) (i.e., effects of Day, Figs. 2 and 4, Additional file 1: Tables S2 and S3).

There were also significant treatment (clipped versus unclipped) X day (days after clipping) interactions for four invertase genes in Col-4 (At3g13790, up-regulation of clipped plants at 1 DAC; At5g11920 and At1g56560, up-regulation of clipped plants at 5 DAC; At1g22650, up-regulation of unclipped plants at 50 % flowering; see Figs. 2, 3 and 4). No treatment X day interactions were observed for L*er* for any of the invertases.

### Knocking out invertase reduces plant compensation for damage

Given that several invertase genes displayed statistically significant changes in expression in response to clipping, we asked whether these genes were involved in the mechanism(s) that promote overcompensation. To do this we quantified the yields of siliques in clipped and unclipped plants harboring single invertase gene knockouts compared with the parent Col-4 line and the undercompensating L*er*.

**Fig. 5:**
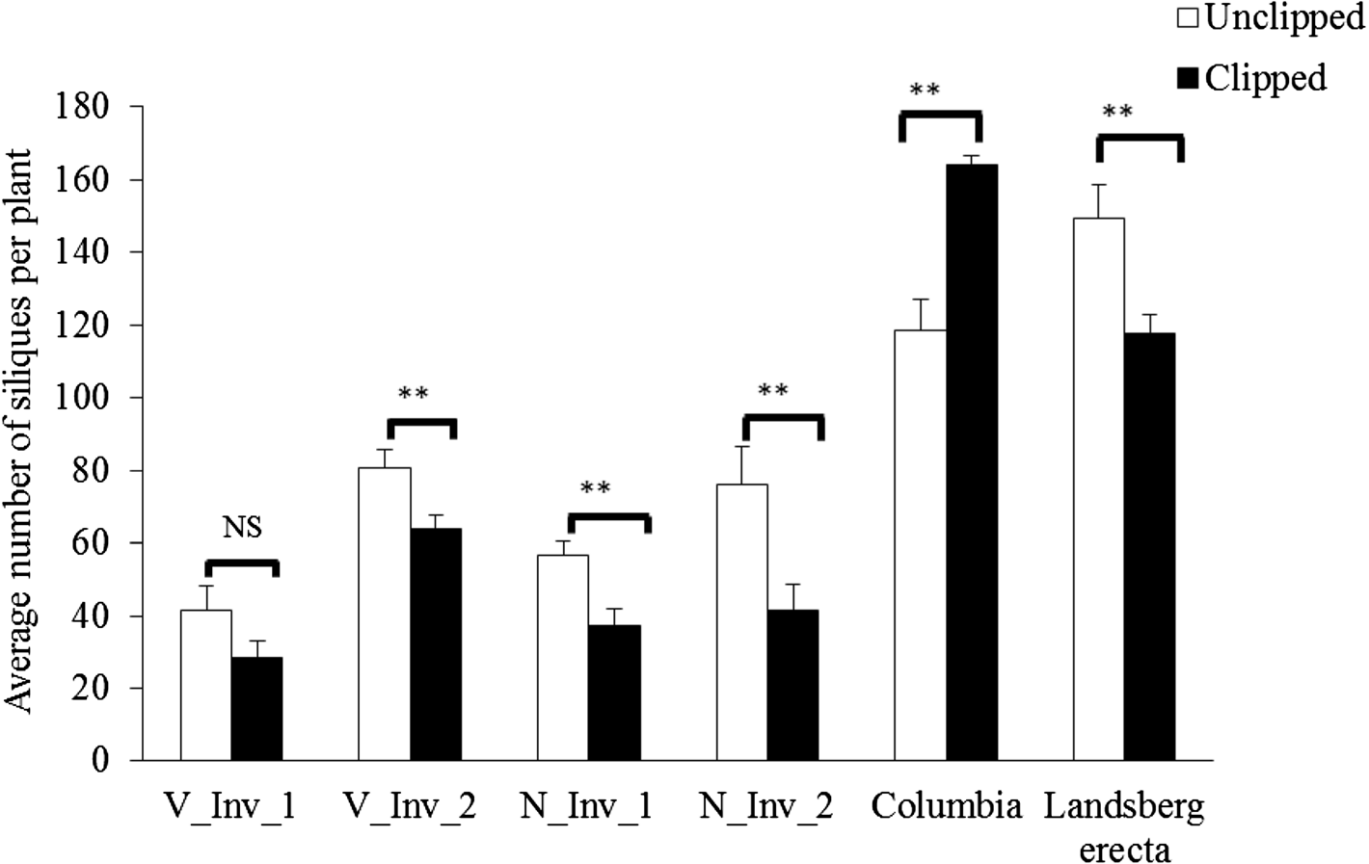
Silique yield for unclipped and clipped T-DNA knockout lines of At1g35580 (SAIL_637_C02 and Wisc450D11, V_Inv_1 and V_Inv_2, respectively) and At4g09510 (SAIL_441_G04 and SAIL_518_D02, N_Inv_1 and N_Inv_2, respectively), the wild type Columbia-4, and Landsberg *erecta*. Asterisks indicate that invertase expression differed significantly between unclipped and clipped plants at * *p* < 0.05 and ** *p* < 0.01

Overall there were significant line (genotypic/knockout; F = 88.3, df = 5, 137, *p* < 0.0001), clipping (F = 9.97, df = 1,137, *p* < 0.0001) and line X clipping effects (F = 8.18, df =5, 137, *p* = 0.002) (Fig. 5). Knocking out the function of the vacuolar invertase (At1g35580) reduced plant compensatory performance, with clipped plants exhibiting an approximately 20 to 32 % reduction in fitness on average relative to unclipped plants, depending on the T-DNA insertion position (equal compensation with a trend toward undercompensation in *VInv_1*: *p* = 0.092, and significant undercompensation in *VInv_2*: *p* = 0.037). Fitness was similarly reduced by approximately 33 to 47 % in clipped plants relative to unclipped plants for the neutral invertase (At4g09510) knockout mutants (with significant undercompensation in both *NInv_1*: *p* < 0.0001, and *NInv_2*: *p* = 0.03). As expected, clipping led to an approximate 28 % increase in fruit production for the wild type Col-4 (significant overcompensation, *p* < 0.0001) and a 20 % decrease in fitness in L*er* upon clipping (significant undercompensation, *p* = 0.003; Fig. 5). It should be noted that unclipped knockout plants had lower height (data not shown) and fitness compared to unclipped Col-4 wild type plants, suggesting that these invertases likely play a role in normal growth and development in addition to regrowth following apical damage.

## Discussion

In this study, we observed a disparate response of invertase gene expression to apical damage in the *A. thaliana* genotypes Col-4 and L*er*. Specifically, nine of twelve invertase isoenzymes assessed were up-regulated (clipped plants vs. unclipped plants) in Col-4 in at least one point in time during regrowth; of these differentially-expressed invertases, all three types were represented (i.e. cell wall, neutral/cytosolic, and vacuolar). The response in gene expression was accompanied by overcompensation for silique production in Col-4, whereas L*er* down-regulated two invertase genes during regrowth and undercompensated. Experimentally knocking out the expression of two invertase isoenzymes caused the Col-4 genetic background to suffer a significant reduction in fitness when damaged; Col-4 with functional invertases overcompensates. Collectively, these results provide direct experimental evidence of the important and previously unrecognized role of invertases in compensation, contributing to our understanding of the mechanisms by which plants respond to, compensate for, and potentially even benefit from apical damage.

Overall, results show differences in plasticity in the expression of invertases following the removal of apical dominance. Col-4, an overcompensating genotype, showed that nine (two vacuolar, two cell wall, and five neutral invertases) of twelve invertase isoenzymes were significantly up-regulated one to five days after the removal of apical dominance whereas L*er*, an undercompensating genotype, showed only a significant decline in two neutral invertases at 15 days post-clipping. These results are consistent with the patterns observed for *G6PD1* in previous studies [23], where it is up-regulated at five days post-clipping in Col-4, likely due, in part, to an increase in glucose fed from invertase isoenzymes into the oxidative pentose phosphate pathway (OPPP), which may then facilitate rapid regrowth, greater biomass accumulation, and ultimately increased fitness. Thus, these results demonstrate a significant timing effect of invertase activity following clipping consistent with the observed differences in the degree of compensation.

Furthermore, there was a general trend toward higher expression at 50 % flowering for both clipped and unclipped plants in five of twelve isoenzymes in the genotype Col-4 and three of twelve in L*er* (see Figs. 2, 3 and 4). These results suggest that Col-4 and to a lesser degree L*er* may up-regulate gene expression over earlier time periods in order to facilitate flower and fruit development. These results are also consistent with the patterns observed for *G6PD1*, showing greater up-regulation at 50 % flowering post-clipping in Col-4 (i.e., with a greater number of invertases up-regulating to supply the added glucose for increased flower and fruit production in the overcompensating genotype Col-4 versus the undercompensating genotype L*er*).

There were few differences in average gene expression (average values of expression across all developmental time points) between clipped and unclipped plants for L*er* or Col-4. One invertase (a neutral invertase, At4g34860) in L*er* and two in Col-4 (a cell wall invertase, Atg12240 and a neutral invertase, Atg22650) showed significant overall cumulative differences in gene expression between clipped and unclipped plants, with unclipped plants in L*er* and Col-4 showing greater overall expression (Additional file 1: Tables S3 and S4). Thus, it is unlikely that differences in compensation can be explained by the average or overall effects of gene expression.

The T-DNA knockout experiments on the two invertase genes, the vacuolar invertase (*VInv_1*, At1g35580) and the neutral invertase (*NInv_1*, At4g09510), and their isoforms confirm their importance in plant growth and fitness in *A. thaliana* following the removal of apical dominance. In three of four cases, fitness was significantly reduced following clipping in the knockout mutants (both T-DNA insertion positions of the neutral invertase and one of the T-DNA insertion positions of the vacuolar invertase) and in the remaining case there was a non-significant trend toward a reduction in fitness (i.e., toward undercompensation; Fig. 5). All four of the mutant knockout lines share the same genetic background as Col-4, therefore the difference in compensatory performance is likely due directly to knocking out the function of the particular invertase. The reduction in overall size/silique production in all four of the mutant lines when unclipped relative to Col-4 is likely due to the role these invertases play in normal plant growth and development in addition to their demonstrated role in compensatory regrowth. Invertases may be particularly important in normal root development, since roots with disrupted vacuolar invertase function are typically shortened relative to wild type roots [32]. Interestingly, because our single invertase knockout approach caused a complete reversal of Col-4’s propensity for overcompensation, there does not appear to be any functional redundancy by the other eleven invertases or by any of the sucrose synthases (it is, however, important to point out that a recent study by Barratt et al. [32] demonstrated that none of the sucrose synthases, of which there are six isoenzymes, are required for normal growth and reproduction). Thus, at least two invertase genes appear to be necessary for normal growth, development and reproduction and, most importantly here, for growth and fitness compensation following apical damage .

From a functional perspective, plants use sucrose and its metabolites glucose and fructose for growth and development. Sucrose is metabolized by invertase (EC 3.2.1.26) to yield glucose and fructose. In spite of location differences (i.e., within the cell wall space, the cytoplasm, and vacuoles), all forms of invertase are enzymatically similar as catalysts of sucrose catabolism, though they do have different functional roles. For example, cell wall invertases are involved in phloem unloading and sink strength, promoting embryo growth, enhanced branching, and flower and pollen development by supplying hexoses to the developing anthers and ovaries [36]. Vacuolar invertases play an important role in the process of cell division essential for seed filling [14, 37, 38], hexose accumulation during fruit set and ripening [39], tissue expansion in tubers [40], and root development [41]. Similarly, neutral invertases are involved in plant growth and development through their involvement in respiration and the biosynthesis of primary and secondary compounds [41 - 43]. The results of our gene knockout experiment indicate that at least for the assessed invertases, individual invertases are not functionally redundant with other invertase isoenzymes from within or between invertase functional groups following apical damage.

## Conclusions

**Fig. 6:**
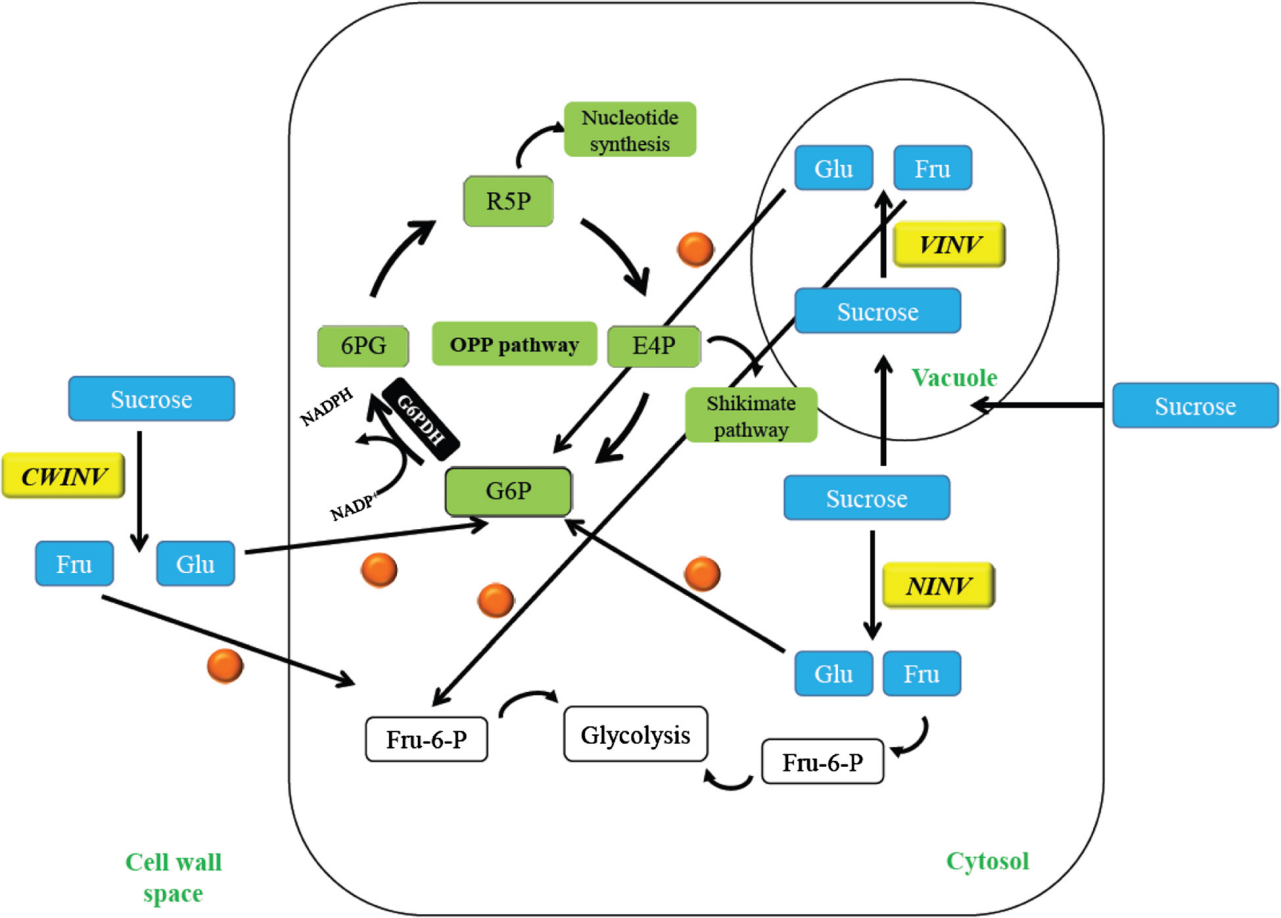
Simplified model representing function of all forms of invertase enzymes—cell wall (CWINV), neutral/cytosolic (NINV) and vacuolar (VINV). Sucrose is hydrolyzed by invertase to yield glucose and fructose. Fructose is phosphorylated by hexokinase (HXK- shown as orange circles) and eventually shunted into the glycolytic pathway. Glucose is similarly phosphorylated by HXK, converting glucose to glucose-6-phosphate (G6P) which is used in the oxidative pentose phosphate pathway (OPPP). GLUCOSE-6-PHOSPHATE DEHYDROGENASE 1 (G6PD1) (EC 1.1.1.49) present in the cytosol oxidizes G6P to yield 6-phosphogluconate (6PG) and in the process reduces NADP to NADPH. NADPH is a required reductant for generalized biosynthesis and assimilatory processes. The 6PG is eventually converted to ribulose-5-phosphate (R5P) and erythrose-4-phosphate (E4P), which are major intermediates to the shikimate pathway and nucleic acid biosynthesis [30]

Though it is clear that genetic variation for fitness compensation exists [17, 20–26, 44], little until recently was known about the genetic underpinnings leading to enhanced growth and reproduction in species exhibiting growth compensation following herbivory. In a previous study [23], we uncovered a key enzyme, *GLUCOSE-6-PHOSPHATE DEHYDROGENASE* (*G6PD1*), that appears to play a significant role in fitness compensation. Here we determined the importance of invertase isoenzymes in the compensatory responses of Col-4 and L*er* accessions of *A. thaliana*. Given that invertases represent one class of enzymes that shunt glucose to activate the OPPP, the results of this study provide a demonstration of the integration between the action of invertases and compensation following damage, presumably through the biosynthetic reactions of the OPPP and downstream processes (Fig. 6). In fact, following the removal of above-ground tissues by herbivory, and thus when plants lack any substantial photosynthetic capacity, the OPPP becomes the primary source of the reductant NADPH in the remaining non-photosynthetic cells for continued biosynthesis and generalized metabolism (including the assimilation of nitrogen into amino acids, fatty-acid synthesis, and antioxidant production) [29]. Intermediates, such as ribose-5-phosphate, are also withdrawn from the OPPP for phenylpropanoid production via the shikimate pathway (Fig. 6) [29, 31].

Apart from fundamental insights gained on the genetic, molecular, and physiological effects on plant responses to damage, understanding the genetic basis of overcompensation (increased seed yield following apical damage) in particular should be of great interest to agriculturists who, through recent advents in genetic technology and selective breeding, might incorporate agronomically important traits into crop plants. For example, ratoon cropping (harvesting the above-ground biomass and allowing the plant to regenerate the following season) is a common practice in rice, sugarcane, and sorghum. By understanding the genetic basis of plant compensation, bioengineering of new cultivars with increased tolerance to damage might, for example, be achieved through the overexpression of genes of large effect. From a broader evolutionary perspective, this study improves our understanding of the mechanisms by which plants respond to, and potentially even benefit from, apical damage.

## Additional file

Additional file 1:
**Table S1.** Primers used for evaluating gene expression of the invertase isoenzymes. **Table S2.** Primers used for genotyping the T-DNA knockout lines. **Table S3.** *P* values of comparisons of gene expression between clipped and unclipped plants in ecotype Columbia-4 at different growth stages following removal of apical dominance. **Table S4.** *P* values of comparisons of gene expression between clipped and unclipped plants in ecotype Landsberg *erecta* at different growth stages following removal of apical dominance. (DOC 106 kb)

## Abbreviations

Col-4: Columbia-4
CWINV: Cell wall invertase
1, 5, 15 DAC: 1, 5, 15 days after clipping
G6PD1: Glucose-6-phosphate dehydrogenase 1
L*er*: Landsberg *erecta*
NINV: Neutral/cytosolic invertase
OPPP: Oxidative pentose phosphate pathway
VINV: Vacuolar invertase

## References

[1] Chew RM (1974). Consumers as regulators of ecosystems: an alternative to energetics. Ohio J Sci.

[2] Dyer MI (1975). The effects of red-winged blackbirds (Agelaius phoeniceus l.) on biomass production of corn grains (Zea mays L.). J Appl Ecol.

[3] Owen DF, Wiegert RG (1976). Do consumers maximize plant fitness?. Oikos.

[4] Whitham TG, Maschinski J, Larson KC, Paige KN, Price PW, Lewinsohn TM, Fernandes GW, Benson WW (1991). Plant responses to herbivory: The continuum from negative to positive and underlying physiological mechanisms. Plant-animal interactions: evolutionary ecology in tropical and temperate regions.

[5] Belsky AJ (1986). Does herbivory benefit plants? A review of the evidence. Am Nat.

[6] Verkaar HJ (1986). When does grazing benefit plants?. Trends Ecol Evol.

[7] Paige KN, Whitham TG (1987). Flexible life history traits: shifts by scarlet gilia in response to pollinator abundance. Ecology.

[8] Vail SG (1992). Selection for overcompensatory plant responses to herbivory: a mechanism for the evolution of plant-herbivore mutualism. Am Nat.

[9] Paige KN (1992). Overcompensation in response to mammalian herbivory: from mutulastic to antagonistic interactions. Ecology.

[10] Paige KN (1994). Herbivory and Ipomopsis aggregata: differences in response, differences in experimental protocol: a reply to Bergelson and Crawley. Am Nat.

[11] Paige KN (1999). Regrowth following ungulate herbivory in Ipomopsis aggregata: geographic evidence for overcompensation. Oecologia.

[12] Anderson LL, Paige KN (2003). Multiple herbivores and coevolutionary interactions in an Ipomopsis hybrid swarm. Evol Ecol.

[13] Maschinski J, Whitham TG (1989). The continuum of plant responses to herbivory: the influence of plant association, nutrient availability, and timing. Am Nat.

[14] Alward R, Joern A (1993). Plasticity and overcompensation in grass responses to herbivory. Oecologia.

[15] Lowenberg GJ (1994). Effects of floral herbivory on maternal reproduction in Sanicula arctopoides (Apiaceae). Ecology.

[16] Lennartsson T, Tuomi J, Nilsson P (1997). Evidence for an evolutionary history of overcompensation in the grassland biennial Gentianella campestris (Gentianaceae). Am Nat.

[17] Weinig C, Stinchcombe JR, Schmitt J (2003). Evolutionary genetics of resistance and tolerance to natural herbivory in Arabidopsis thaliana. Evolution.

[18] Rautio P, Huhta AP, Piippo S, Tuomi J, Juenger T, Saariet M (2005). Overcompensation and adaptive plasticity of apical dominance in Erysimum strictum (Brassicaceae) in response to simulated browsing and resource availability. Oikos..

[19] Stowe KA, Marquis RJ, Hochwender CG, Simms EL (2000). The evolutionary ecology of tolerance to consumer damage. Annu Rev Ecol Syst.

[20] Mauricio R, Rausher MD, Burdick DS (1997). Variation in the defense strategies of plants: are resistance and tolerance mutually exclusive?. Ecology.

[21] Tiffin P, Rausher MD (1999). Genetic constraints and selection acting on tolerance to herbivory in the common morning glory Ipomoea purpurea. Am Nat.

[22] Juenger T, Bergelson J (2000). Factors limiting rosette recruitment in scarlet gilia, Ipomopsis aggregata: seed and disturbance limitation. Oecologia.

[23] Siddappaji MH, Scholes DR, Bohn M, Paige KN (2013). Overcompensation in response to herbivory in Arabidopsis thaliana: the role of glucose-6-phosphate dehydrogenase and the oxidative pentose-phosphate pathway. Genetics.

[24] Scholes DR, Siddappaji MH, Paige KN (2013). The genetic basis of overcompensation in plants: a synthesis. Int J Mod Bot.

[25] Scholes DR, Paige KN (2014). Plasticity in ploidy underlies plant fitness compensation to herbivore damage. Mol Ecol.

[26] Scholes DR, Paige KN (2011). Chromosomal plasticity: mitigating the impacts of herbivory. Ecology.

[27] Scholes DR, Paige KN (2015). Plasticity in ploidy: a generalized response to stress. Trends Plant Sci.

[28] Fotopoulos V (2005). Plant invertases: structure, function and regulation of a diverse enzyme family. J Biol Res.

[29] Kruger NJ, von Schaewen A (2003). The oxidative pentose phosphate pathway: structure and organisation. Curr Opin Plant Biol.

[30] Eicks M, Maurino V, Knappe S, Flügge UI, Fischer K (2002). The plastidic pentose phosphate translocator represents a link between the cytosolic and the plastidic pentose phosphate pathways in plants. Plant Physiol.

[31] Scharte J, Schön H, Tjaden Z, Weis E, von Schaewen A (2009). Isoenzyme replacement of glucose-6-phosphate dehydrogenase in the cytosol improves stress tolerance in plants. Proc Natl Acad Sci U S A.

[32] Barratt DHP, Derbyshire P, Findlay K, Pike M, Wellner N, Lunn J (2009). Normal growth of Arabidopsis requires cytosolic invertase but not sucrose synthase. Proc Natl Acad Sci U S A.

[33] Pfaffl MW (2001). A new mathematical model for relative quantification in real-time RT–PCR. Nucleic Acids Res.

[34] Lehtilä K, Boalt E. The use and usefulness of artificial herbivory in plant-herbivore studies. In: Insects and Ecosystem Function, Weisser, WW, Siemann E (Eds.) Ecological Studies 2004;173:257-75.

[35] Hjältèn J. Simulating herbivory: Problems and possibilities. In: Insects and Ecosystem Function, Weisser, WW, Siemann E (Eds.) Ecological Studies. 2004;173:243-56.

[36] Zhang XY, Wang XL, Wang XF (2006). A shift of phloem unloading from symplasmic to apoplasmic pathway is involved in developmental onset of ripening in grape berry. Plant Physiol.

[37] Ruan YL, Jin Y, Yang YJ (2010). Sugar input, metabolism, and signaling mediated by invertase: roles in development, yield potential, and response to drought and heat. Mol Plant.

[38] Ruan YL (2012). Signaling role of sucrose metabolism in development. Mol Plant.

[39] Jin Y, Ni D-A, Ruan YL (2009). Posttranslational elevation of cell wall invertase activity by silencing its inhibitor in tomato delays leaf senescence and increases seed weight and fruit hexose level. Plant Cell.

[40] Ross HA, Davies HV, Burch LR, Li J, Wu Y, Wu P (1994). Developmental changes in carbohydrate content and sucrose degrading enzymes in tuberising stolons of potato (Solanum tuberosum). Physiol Plant.

[41] Lou Y, Gou J-Y, Xue H-W (2007). PIP5K9, an Arabidopsis phosphatidylinositol monophosphate kinase, interacts with a cytosolic invertase to negatively regulate sugar-mediated root growth. Plant Cell.

[42] Jia L, Zhang B, Mao C (2008). OsCYT-INV1 for alkaline/neutral invertase is involved in root cell development and reproductivity in rice (*Oryza sativa* L.). Planta.

[43] Welham T, Pike J, Horst I (2009). A cytosolic invertase is required for normal growth and cell development in the model legume, Lotus japonicus. J Exp Bot.

[44] Weinig C, Stinchcombe JR, Schmitt J (2003). QTL architecture of resistance and tolerance traits in Arabidopsis thaliana in natural environments. Mol Ecol.

