# Supplementary material for: The role of invertases in plant compensatory responses to simulated herbivory
Source: BMC Plant Biol. 2015 Nov 16;15:278. doi: 10.1186/s12870-015-0655-6 (PMC4647499; doi:10.1186/s12870-015-0655-6)
Supplement: Additional file 1: — Table S1. Primers used for evaluating gene expression of the invertase isoenzymes. Table S2. Primers used for genotyping the T-DNA knockout lines. Table S3. P values of comparisons of gene expression between clipped and unclipped plants in ecotype Columbia-4 at different growth stages following removal of apical dominance. Table S4. P values of comparisons of gene expression between clipped and unclipped plants in ecotype Landsberg erecta at different growth stages following removal of apical dominance. (DOC 106 kb) [file 12870_2015_655_MOESM1_ESM.doc]

**Additional file 1: Table S1: Primers used for evaluating gene expression of the invertase isoenzymes. CW-inv, N-inv and V-inv represents cell wall-, neutral- and cytosolic- invertases, respectively.**

| **Sl. No.** | **Primer Name** | **Invertase type** | **Sequence** |
| --- | --- | --- | --- |
| 1 | 1g_12240F | CW-Inv | TTGCCAATCTCCGCCAGAGAAGAAGAACCA |
|  | 1g_12240R |  | GTACGGGAGAGAGCACGGACAGCTTCGTCA |
| 2 | 1g_55120F | CW-Inv | AAGCTTCTTCTCATCAAGATCTCAACCAAC |
|  | 1g_55120R |  | TCAAAACTGATGATTAGGTGAGCGTGACTC |
| 3 | 3g_13790F | CW-Inv | GACTTTGGTTATTGCTCACGTTACTTATTG |
|  | 3g_13790R |  | CAACGTACTTATTGCTGTTTTGACGGGCTT |
| 4 | 5g_11920F | CW-Inv | ATGGAACAGAATCTTCTTCAAACCGCTGTG |
|  | 5g_11920R |  | AAGTTGGGTTTGAATTGATTTGGGCATTTC |
| 5 | 1g_22650F | N-Inv | GTAAACTCTTCAAGCTCTATATCCGACCTA |
|  | 1g_22650R |  | AGTCCAAGAATAAGATCTCTTAATGACAGG |
| 6 | 1g_56560F | N-Inv | GAGCGCAATCTATCTCCTCCGCAAAATTTC |
|  | 1g_56560R |  | TCGAACAAGAATCTGAGTCTTGGCAGCGAC |
| 7 | 3g_06500F | N-Inv | ATGAACAGTAGAAGCTGTATCTGTGTCTCT |
|  | 3g_06500R |  | GATCTTGGAGGTGTGAAACGCGAACACTTG |
| 8 | 4g_34860F | N-Inv | AGTTTTAATCTGAGTGTAGATGTGAATCAG |
|  | 4g_34860R |  | CAAGTCCAGGAGTTGGATCTTCTCATAACG |
| 9 | 5g_22510F | N-Inv | TGGCAGCTTCAGAAACAGTTCTACGTGTTC |
|  | 5g_22510R |  | TTGAGCTTTTTTGGGTCCTCTTGTCCTCCT |
| 10 | 4g_09510F | N-Inv | CATAAAGAACCATTGGTGCTAAGAGTTGAA |
|  | 4g_09510R |  | CAAGTCCATGAAGCAGATCTCTTGATAACA |
| 11 | 1g_35580F | V-Inv | ATGGAAGGTGTTGGACTAAGAGCTGTAGGA |
|  | 1g_35580R |  | AGTTGTGGCCAAGACGCAGATCGCTTGATG |
| 12 | 1g_62660F | V-Inv | ACCCGTCACGTCCCTACAAGATCCATTATC |
|  | 1g_62660R |  | GTGCTGGAAGGAACACCGAGATCGTCTGAA |
| 13 | 4G_27960F | Ubiquitin | TCACAATTTCCAAGGTGCTGC |
|  | 4G_27960R |  | TCATCTGGGTTTGGATCCGT |

**Additional file 1: Table S2: Primers used for genotyping the T-DNA knockout lines obtained from *Arabidopsis* Biological Resource Center. The CW-inv, N-inv and V-inv represent cell wall-, neutral- and vacuolar- invertase, respectively.**

| **Primer Name** | **Sequence** |
| --- | --- |
| **LP_V_inv_1** | GTCTCCCTGTCTTAATGCACG |
| **RP_V_inv_1** | CTTCATGGCTTTGAGATCTGC |
| **LB_V_inv_1** | TAGCATCTGAATTTCATAACCAATCTCGATACAC |
| **LP_V_inv_2** | CAATCGACCAAATGAGTGAGG |
| **RP_V_inv_2** | CGCTAGACCTAGCCATTAGGG |
| **LB_V_inv_2** | TAGCATCTGAATTTCATAACCAATCTCGATACAC |
| **LP_N_inv_1** | TTCTCTCGTGACTCAATTGCC |
| **RP_N_inv_1** | TCCATGAGAACGAACCAGATC |
| **LB_N_inv_1** | ATTTTGCCGATTTCGGAAC |
| **LP_N_inv_2** | TGGGAGCCACATAATTCAAAG |
| **RP_N_inv_2** | CATAGCCAGTCGATAACTCGC |
| **LB_V_inv_2** | AACGTCCGCAATGTGTTATTAAGTTGTC |

**Additional file 1: Table S3: P values of comparisons of gene expression between clipped and unclipped plants in ecotype Columbia-4 at different growth stages following removal of apical dominance (one day before clipping not shown) . The p values for 1, 5, 15 days after clipping (DAC) and 50% flowering were calculated using two sample t-tests. The p values for treatment, days and treatment × days were calculated using a 2-way ANOVA (including one day before clipping). Cell wall, neutral and vacuolar invertases are designated as CW-inv, N-inv and V-inv, respectively. Asterisks represent significance levels of * p<0.05 and ** p<0.01. Plus (+) and minus (–) signs designate significant up- or down-regulation, respectively, upon clipping for the given gene and time point.**

| **Columbia-4** | **Invertase class** | **1 DAC** | **5 DAC** | **15 DAC** | **50% flowering** | **Treatment** | **Days** | **Treatment × Days** |
| --- | --- | --- | --- | --- | --- | --- | --- | --- |
| At1g35580 | V_inv_1 | 0.07 ^NS^ | 0.05 * + | 0.96 ^NS^ | 0.56 ^NS^ | 0.93 | 0.12 | 0.58 |
| At1g62660 | V_inv_2 | 0.89 ^NS^ | 0.02 * + | 0.37 ^NS^ | 0.39 ^NS^ | 0.36 | 0.46 | 0.22 |
| At1g12240 | CW_inv_1 | 0.002 ** – | 0.55 ^NS^ | 0.15 ^NS^ | 0.14 ^NS^ | 0.03 * | 0.001** | 0.08 |
| At1g55120 | CW_inv_2 | 0.95 ^NS^ | 0.19 ^NS^ | 0.87 ^NS^ | 0.73 ^NS^ | 0.39 | 0.003 ** | 0.33 |
| At3g13790 | CW_inv_3 | 0.05 * + | 0.17 ^NS^ | 0.28 ^NS^ | 0.85 ^NS^ | 0.23 | 0.02 * | 0.02 ** |
| At5g11920 | CW_inv_4 | 0.23 ^NS^ | 0.05 * + | 0.73 ^NS^ | 0.61 ^NS^ | 0.10 | 0.03 * | 0.05 * |
| At1g22650 | N_inv_1 | 0.28 ^NS^ | 0.33 ^NS^ | 0.33 ^NS^ | 0.29 ^NS^ | 0.013 ** | 0.002 ** | 0.048 * |
| At1g56560 | N_Inv_2 | 0.24 ^NS^ | 0.02 * + | 0.91 ^NS^ | 0.59 ^NS^ | 0.07 | 0.20 | 0.006 ** |
| At1g06500 | N_inv_3 | 0.65 ^NS^ | 0.01 ** + | 0.46 ^NS^ | 0.65 ^NS^ | 0.82 | 0.001 ** | 0.72 |
| At4g09510 | N_inv_4 | 0.48^NS^ | 0.05 * + | 0.54 ^NS^ | 0.51 ^NS^ | 0.30 | 0.007 ** | 0.12 |
| At4g34860 | N_inv_5 | 0.79 ^NS^ | 0.008 ** + | 0.38 ^NS^ | 0.39 ^NS^ | 0.36 | 0.46 | 0.45 |
| At5g22510 | N_inv_6 | 0.59 ^NS^ | 0.03 * + | 0.34 ^NS^ | 0.38 ^NS^ | 0.31 | 0.51 | 0.45 |

**Additional file 1: Table S4: P values of comparisons of gene expression between clipped and unclipped plants in ecotype Landsberg *erecta* at different growth stages following removal of apical dominance (one day before clipping not shown). The p values for 1, 5, 15 days after clipping (DAC) and 50% flowering were calculated using two sample t-tests. The p values for treatment, days and treatment × days were calculated using a 2-way ANOVA (including one day before clipping). Cell wall, neutral and vacuolar invertases are designated as CW-inv, N-inv and V-inv, respectively. Asterisks represent significance levels of * p<0.05 and ** p<0.01. Minus (-) signs designate significant down-regulation upon clipping for the given gene and time point (no genes were significantly up-regulated at any time point).**

| **Landsberg *erecta*** | **Invertase class** | **1 DAC** | **5 DAC** | **15 DAC** | **50% flowering** | **Treatment** | **Days** | **Treatment × Days** |
| --- | --- | --- | --- | --- | --- | --- | --- | --- |
| At1g35580 | V_inv_1 | 0.41 ^NS^ | 0.50 ^NS^ | 0.34 ^NS^ | 0.71 ^NS^ | 0.76 | 0.52 | 0.68 |
| At1g62660 | V_inv_2 | 0.37 ^NS^ | 0.92 ^NS^ | 0.39 ^NS^ | 0.21 ^NS^ | 0.49 | 0.42 | 0.39 |
| At1g12240 | CW_inv_1 | 0.38 ^NS^ | 0.54 ^NS^ | 0.25 ^NS^ | 0.85 ^NS^ | 0.39 | 0.001** | 0.82 |
| At1g55120 | CW_inv_2 | 0.30 ^NS^ | 0.70 ^NS^ | 0.82 ^NS^ | 0.96 ^NS^ | 0.46 | 0.002 ** | 0.67 |
| At3g13790 | CW_inv_3 | 0.39 ^NS^ | 0.09 ^NS^ | 0.66 ^NS^ | 0.28 ^NS^ | 0.47 | 0.02 * | 0.26 |
| At5g11920 | CW_inv_4 | 0.31 ^NS^ | 0.73 ^NS^ | 0.41 ^NS^ | 0.40 ^NS^ | 0.07 | 0.01 * | 0.65 |
| At1g22650 | N_inv_1 | 0.26 ^NS^ | 0.83 ^NS^ | 0.54 ^NS^ | 0.91^NS^ | 0.10 | 0.007 ** | 0.47 |
| At1g56560 | N_inv_2 | 0.39 ^NS^ | 0.95 ^NS^ | 0.04 * – | 0.70 ^NS^ | 0.26 | 0.30 | 0.52 |
| At1g06500 | N_inv_3 | 0.93 ^NS^ | 0.99 ^NS^ | 0.18 ^NS^ | 0.58 ^NS^ | 0.37 | 0.10 | 0.65 |
| At4g09510 | N_inv_4 | 0.25 ^NS^ | 0.22 ^NS^ | 0.02 * – | 0.33 ^NS^ | 0.09 | 0.39 | 0.51 |
| At4g34860 | N_inv_5 | 0.22 ^NS^ | 0.66 ^NS^ | 0.18 ^NS^ | 0.51 ^NS^ | 0.03 * | 0.03 * | 0.74 |
| At5g22510 | N_inv_6 | 0.41 ^NS^ | 0.21 ^NS^ | 0.20 ^NS^ | 0.53 ^NS^ | 0.33 | 0.03 * | 0.54 |
